# Risk Reduction Research Initiative: A National Community–Academic Framework to Improve Health and Social Outcomes

**DOI:** 10.1089/pop.2018.0099

**Published:** 2019-08-02

**Authors:** Mark Redding, John Hoornbeek, Bernard P. Zeigler, Michael Kelly, Sarah Redding, Lynn Falletta, Karen Minyard, Edward Chiyaka, David Bruckman

**Affiliations:** ^1^ Rebecca D. Considine Research Institute, Akron Children's Hospital, Mansfield, Ohio.; ^2^ Center for Public Policy and Health, College of Public Health, Kent State University, Kent, Ohio.; ^3^ RTSync. Corp, Potomac, Maryland.; ^4^ Rebecca D. Considine Research Institute, Akron Children's Hospital, Akron, Ohio.; ^5^ Pathways Community HUB Institute, Mansfield, Ohio.; ^6^ Department of Public Management and Policy, Georgia Health Policy Center, Atlanta, Georgia.

**Keywords:** health and socioeconomic outcomes, social determinants of health, value based payment, modifiable factors of risk, weight of impact, care coordination

## Introduction

The American health care system is costly and underperforms compared to health care systems in other developed countries.^1^ In our view, one reason for this poor performance is that our health system does not identify and address the *full range of risks* affecting health and socioeconomic outcomes. As our health care system is transitioning from a fee-for-service to a value-based payment approach, there are opportunities to improve effectiveness and reduce costs. To achieve better health outcomes, our health system should comprehensively address medical, social, and behavioral factors of risk.^2^ If this is done, policy makers and practitioners will be able to prioritize investments and interventions more effectively to address the most important risks contributing to health and other important socioeconomic outcomes.

In this commentary, we present a research initiative framework to inform beneficial transformations in the American health and social service systems. It is predicated on the idea that there is a need for a more comprehensive approach to identify and address important individually modifiable factors of risk – particularly for at-risk individuals. The framework seeks to guide research to improve our understanding of risk factors, their interrelationships, and their relative impact on a range of specific outcomes, including medical outcomes such as disease control and hospitalization, as well as other critical socioeconomic outcomes such as school performance and employment. Building knowledge in these areas, we argue, is needed to increase the ability of policy makers and health professionals to improve health and other outcomes through more effective and efficient interventions, investments, and payment models.

## The Problem

Spending per capita on health care in the United States is the highest in the developed world, and benchmark outcomes such as infant mortality and life expectancy remain among the worst across developed countries.^1^ How can the United States have some of the worst health outcomes in the developed world while spending the most per capita on a system whose purpose is to improve those outcomes?

We hypothesize that greater knowledge regarding risk factors, the most critical actionable components of improved health, will contribute to decreasing the discrepancy between US spending levels and outcomes. Medical research on health outcomes often focuses on singular factors of risk being addressed through specific and singular medical interventions, even though social and behavioral factors of risk have been scientifically confirmed to be critical to health outcomes.^2^ Similarly, our health and social service system often relies on public programs that are focused on singular purposes and/or limited risk factors within siloed operational structures.^3^

As a result of these and other sources of service fragmentation, effective care coordination is often lacking – particularly for at-risk populations. For example, an expectant mother may be at risk because she lacks housing, does not have access to prenatal care, and is depressed. Unless all 3 of these risk factors are addressed, she and her child remain at significant risk for poor health outcomes. The factors are interdependent, as she may not be able to prioritize prenatal care unless her depression and the housing factors are addressed. Even if she gets appropriate prenatal care, without treatment for depression and housing access, she may remain in significant stress, a known risk for pregnancy outcomes.^4,5^

This example highlights just a few of the many risk factors that work individually and collectively to impact health and socioeconomic outcomes. The American health and social services system is not well equipped to address the complexities of this situation. At-risk individuals with complex interconnected health and social risk factors may have to find resources to travel to multiple agencies and complete multiple lengthy applications to gain access to the services they need, and – even then – may not receive the kind of coordinated care they need to reduce their priority risks.

## Building Evidence to Address the Problem

This research initiative seeks to develop knowledge on medical, social, and behavioral risk factors to help practitioners and policy makers make more informed decisions to improve health and socioeconomic outcomes. For example, in working to build a national health strategy, the Secretary of Health and Human Services may ask which is more important and impactful on health outcomes, lacking health care access or homelessness, and what is the impact when both factors occur together? The relationship between these factors and their combined impact is unknown. We lack quantitative information addressing the relative impact of both singular risks and the combined effects of multiple risk factors (potentially crossing medical, social, and behavioral factors of risk) on health and socioeconomic outcomes. The ability to assign quantitative values to a single risk and groups of risks would allow key decision makers to design more effective interventions and to allocate resources in ways that target risk factors that have the highest likelihood of achieving positive health outcomes.

In this regard, payment models also are relevant to this research initiative. Pay for value has been implemented in health care and is becoming a major aim of the health care payment system. However, only a marginal value has been demonstrated in certain pay-for-value approaches to improve outcomes and reduce cost.^6^ To date, pay-for-value systems most often focus almost completely on medical factors of risk.^7^ The inclusion of a broader and more quantitative assessment of risks would be helpful to target payments toward factors that are most likely to improve outcomes and value.

Proprietary business-related analyses within insurance, hospital systems, and related efforts have realized the benefits of quantitative measures of risk factors.^8^ In these sectors, the development of risk scores is related to knowing the relative impact of specific factors of risk, and risk scores are used to reflect a compilation of individual and/or combined risks to manage populations in relation to services, costs of care, and intensities of services provided.

The information achieved by these business-related initiatives is potentially useful to the strategic development of public health and social service policies and practices, yet is not publicly accessible because of its development and use within privately funded initiatives. It would be valuable to draw insight from these private sector approaches to conduct research and produce expanded quantitative and qualitative information on key characteristics of risk factors and successful risk reduction initiatives to enable public policy makers and other key decision makers to improve health and reduce costs. Because current proprietary resources often do not have information on social determinants of health, this initiative substantially expands on this critical component in a more comprehensive and publicly available evaluation of risk factors and ways they can be addressed.

## Research Potential and Application

Individually modifiable factors of risk represent critical components in medical, social, and behavioral health systems of care. Performing care coordination and direct service interventions that document the identification and mitigation of critical risk factors can provide measurable ways to define accountable work products and guide payment systems to focus on outcomes.

Ideally, care coordination is the comprehensive assessment of an individual's risk factors coupled with the identification and connection to community services required to address her/his needs and mitigate her/his risks. In reality, care coordination is often fragmented and provided by multiple independent programs within communities, each focused on specific factors of risk. In this context, coordinated strategies are necessary to ensure that priority risks are addressed, avoid service duplication, and enable comprehensive measures of impact.^9^

Fortunately, in this context, care coordination and direct services that effectively identify and address social risks (eg, housing, food, clothing, education), medical risks (eg, access to primary and specialty care, medication access), and behavioral health risks (eg, depression, social isolation) now are being provided through a nationally Certified Pathways Community HUB model, which draws on Lean production and related business approaches. The HUB model also includes engagement of culturally connected community members – or community health workers (CHWs) – who serve as care coordinators. With the help of CHWs, certified HUBs are demonstrating risk reduction progress and improvements in outcomes.^10^

In the HUB model, payments to CHWs reimburse confirmed risk factor reductions that occur when “Pathways” reflecting identified risks and steps needed to address them are completed by their clients. By tying payment directly to confirmed mitigation of risk factors within a comprehensive care framework, certified HUBs can reduce risks through coordinated provision of health and social services.

The certification of Pathways Community HUBs through the Rockville Institute, and its standardization of the Pathways nationally across HUBs, demonstrate that risk-based care coordination and payment systems are feasible in the United States. The HUB model has been used for 20 years and more than 30 community HUB programs now exist nationally. Funders supporting these HUBs include Medicaid managed care, departments of health, departments of social service, private foundations, community mill levies, United Way, churches, and others. Through these HUBs, funders realize a direct link between their payments and confirmed steps to reduce risks. Given this array of current funding applications, it seems likely that the HUB model can be integrated with a range of health and social service funding sources.

## Research Aims

To improve health outcomes and increase the value of care, we need to work toward a comprehensive and quantitative accounting of risks, models of care coordination that are comprehensive in scope and span across domains of risk (medical, social, and behavioral), and payment structures that reward risk reduction and improved outcomes. Research is necessary in all these areas to determine optimal approaches to improve the effectiveness and value of health care and social service delivery.

A research thrust focused on risk identification, quantification, and reduction is needed to address the requirements outlined. By building stronger understandings of individually modifiable factors of risk that may work together in their impact on outcomes, this kind of research can better support programming and payment strategies to improve outcomes. We suggest that a framework for such a thrust can be constructed on the basis of the following research aims:

1. Aim #1 – Identify significant individually addressable risk factors by age group category.
2. Aim #2 – Develop weight of impact estimates of the relative impacts of individual risk factors (identified through Aim 1 above), as compared to other risk factors. Weights of impact may be represented in terms of relative weights, probabilities, risk, or additive cost. The major outcomes (dependent variables) on which the risk factor exerts its impact are expected to include examples such as:
  - Medical – Emergency room use, hospitalization, disease control and prevention outcomes, and cost of care;
  - Social – School performance, employment and economic success; and
  - Behavioral health – Parenting attention deficit disorder and depression.
3. Aim #3 – Create estimates of the weights of impact of specific combinations of risk factors on major outcomes, as interactions among risk factors may exert influences on outcomes that are not accounted for by individual risk factors (identified through Aim #1).
4. Aim #4 – Conduct interventional analyses to determine if outcomes and costs of care change when risk factors are identified and addressed, both individually and in combination.
5. Aim #5 – Publish and provide critical programming and payment actionable information to policy makers, payers, programs, research programs, hospital systems, HUBs, and others.

Research consistent with various elements of the framework described is being conducted by the authors of this commentary and others.

Our hope is that this publication provides an actionable framework, amplifying thinking and research motivated and structured by a lens of risk and risk reduction. This work can allow us to better understand and effectively address medical, social, and behavioral health risk reduction programming and payments, thus contributing to a beneficial transformation of the US health and social service system, to produce better outcomes.
